# The impact of *in-situ* urbanization on residents’ health: evidence from CFPS

**DOI:** 10.3389/fpubh.2025.1588944

**Published:** 2025-06-18

**Authors:** Wei Wu, Shuoxuan Zhang, Lei Zhao, Jiawei Gao

**Affiliations:** ^1^Business School of Jiangsu University of Science and Technology, Zhangjiagang, China; ^2^Yangtze River Delta Social Development Research Center, Jiangsu University of Science and Technology, Zhangjiagang, China; ^3^School of Economics and Management, Southeast University, Nanjing, China; ^4^Ministry of Agriculture and Rural Affairs of the People's Republic of China, Beijing, China; ^5^National School of Development and Policy, Southeast University, Nanjing, China

**Keywords:** *in-situ* urbanization, health, difference-in-differences, household income, CFPS

## Abstract

This study investigates the health effects of in-situ urbanization utilizing panel data from the China Family Panel Studies (CFPS) tracking surveys of 2010, 2012, and 2014. Employing a difference-in-differences (DID) model, the analysis reveals a statistically significant positive impact of in-situ urbanization on individual health. Specifically, individuals subjected to in-situ urbanization policies exhibit a 6.7% reduction in the reported incidence of physical discomfort within the preceding two weeks. Heterogeneity in the health effects of in-situ urbanization is observed across demographic strata, including gender, age, and educational attainment. Notably, the health-enhancing effects are more pronounced for female respondents compared to their male counterparts. Furthermore, a discernible age-related disparity is evident, with individuals below the age of 60 demonstrating significant health improvements, while those aged 60 and above do not manifest statistically significant health gains. In terms of educational attainment, individuals with educational levels below high school experience a greater magnitude of health improvement attributable to in-situ urbanization. The mechanism through which in-situ urbanization improves individual health is mediated by an increase in household income. Structurally, this income augmentation is characterized by an increase in operating and property income, coupled with a concomitant decrease in transfer income. Consequently, the elevation of operating and property income emerges as a salient intermediate mechanism facilitating the health improvement effects of in-situ urbanization.

## 1 Introduction

Urbanization is a global phenomenon (1). Since the Industrial Revolution, many countries worldwide have transitioned from agrarian-based rural societies to industrial and service-oriented urban societies (46). As a historical process of human societal evolution, urbanization follows common patterns across countries, yet it manifests differently in various periods and countries (2). Urbanization in developed countries has almost reached maturity, and thus the focus of urbanization has shifted toward developing countries. In particular, China’s unique path to urbanization has attracted widespread attention in the international academic community (3).

Prior to the reform and opening-up, rural areas in China were largely excluded from industrialization and urbanization processes (4). Since the late 1970s, with rapid industrial development and the transfer of surplus agricultural labor, local governments have actively established new urban centers in rural areas in the form of administrative towns to promote township enterprises. This led to a sharp increase in the number of towns in China. The “Notice on the Report on Adjusting the Standards for Town Establishment” issued by the State Council in 1984 [Guofa (1984) No. 165]^1^ modified the criteria for administrative towns to include all county-level government locations and townships with a non-agricultural population exceeding 2,000. By the end of 2021, the number of administrative towns reached 21,322, nearly 10 times that of 1984. During the same period, the number of urban districts also increased significantly. The increase in administrative towns and the expansion of existing urban spaces have become the main drivers of urban population growth for some time. The urban population share has steadily increased and, in 2011, it first surpassed the rural population. By the end of 2022, the urbanization rate in China (the proportion of urban residents in the total population) had reached 65%.

In practice, due to differences in location, factors, and economic development conditions across regions, the paths to in-situ urbanization vary. However, contrary to the common view that migration and rural–urban mobility play a leading role in the urbanization process in many developing countries, an important feature of China’s urbanization is the emergence and development of in-situ urbanization driven by local elites (5–7). In-situ urbanization refers to the reclassification of rural–urban regions led by reputable local elites through resource integration, the development of non-agricultural industries, and infrastructure building (48). Moreover, the growth of urban populations due to in-situ urbanization has become a significant component of overall urban population growth (8–10).

In-situ urbanization transforms rural areas into urban or quasi-urban regions, with little to no geographic migration of farmers (11). This population shift toward an urban lifestyle without physical migration reflects the evolving concept of urbanization as economic and social development progresses, specifically the transition from a rural way of life to an urban one. This transformation process includes the aggregation of factors of production such as labor, technology, and capital, as well as changes in production and living styles, consumption patterns, and ideologies, along with the construction and improvement of urban infrastructure and public services. This trend is widely present across the country, especially in the southeastern coastal regions. The development model of the southeast coast is continually evolving, with the industrial structure gradually adjusting from traditional “labor-intensive” to “innovation-driven” and “capital-intensive” development. This has increased the demand for high-quality labor in the southeast coast while reducing the absorption capacity for general labor from rural areas.

In 2022, the National Development and Reform Commission released the “14th Five-Year Plan for New-type Urbanization,” which provides a reference for establishing a localized urbanization development system. This plan proposes optimizing urbanization spatial layout and forms, guiding the development of small towns, and promoting a unified planning design for urban and rural areas. It also emphasizes extending public services and infrastructure to rural areas and promoting local employment and entrepreneurship for rural labor. Aligned with the goals of the 14th Five-Year Plan, this study examines the health impacts of in-situ urbanization, providing evidence to inform policies aimed at maximizing the health benefits of this process.

China not only has a dual urban–rural economic structure but also a segregated household registration system, so one standard for an individual’s urbanization is the transformation of agricultural household registration to non-agricultural household registration, known as “village-to-residence” (village-to-township transition). The urbanization of the agricultural migrant population involves more than just a change in household registration; it also entails equal treatment in areas such as employment, housing, education, pensions, and healthcare (12). Furthermore, changes in economic systems, social structures, and psychological and social consciousness are crucial in this process (13). Crucially, as rural workers transition from agricultural to non-agricultural employment after urbanization, their wage income becomes a steadily rising source of their total income, contributing to increased earnings (14), along with property income derived from land expropriation and house demolition (15). Understanding these income dynamics is essential for policies seeking to support residents’ economic well-being during in-situ urbanization.

Against this background, this paper will focus on the “village-to-residence” transition and explore the health impact of in-situ urbanization from a micro perspective. Examining the health effects of this transition directly informs policies aimed at optimizing the design and implementation of in-situ urbanization strategies. It will examine the potential mechanisms through which in-situ urbanization affects health, providing suggestions and references for further advancing in-situ urbanization. For example, by analyzing the heterogeneous health effects across gender, age, and education levels, this research can help policymakers design targeted interventions to ensure that the benefits of in-situ urbanization are equitably distributed.

Grossman (50) pioneering work paved the way for the rapid development of health economics, revealing that health is influenced by various factors such as healthcare, economic conditions, and education level. Studies show that healthcare services significantly improve health status, while the relationship between income and health is complex and varies by region and conditions. Education improves health by increasing knowledge and income (16). Poor habits and lifestyles harm health, while healthy behaviors and environments benefit health (17). Other factors, such as occupation and social structure, also influence health. Urbanization affects all these factors to varying degrees, and thus it undoubtedly impacts individual health.

For a long time, the impact of urbanization on health has been extensively studied. However, due to data limitations and the existence of health migration effects, most existing research treats urbanization as an outcome, lacking a comparison of individual health before and after urbanization. Studies specifically focusing on in-situ urbanization are even fewer. Therefore, this paper approaches the topic from the perspective of in-situ urbanization, comparing individual health before and after in-situ urbanization and exploring the potential mechanisms through which in-situ urbanization affects health. This research will enhance the explanatory power of social-ecological theories concerning the health effects of urbanization, filling gaps in existing theoretical frameworks.

Moreover, most existing studies on the health effects of in-situ urbanization rely on cross-sectional data. In fact, health, as a reflection of an individual’s physical and mental condition, is not static; it changes as living environments evolve. Thus, longitudinal data is necessary to observe changes in individual health. Therefore, an additional contribution of this paper is the use of CFPS tracking data from 2010, 2012, and 2014 to study the health impact of in-situ urbanization from a longitudinal perspective.

Using a quasi-natural experiment framework, the study treats the “village-to-residence” transitions occurring between 2011 and 2013 as a natural experiment, dividing the samples into treatment and control groups. The difference-in-differences (DID) method is used to estimate the treatment effects of in-situ urbanization on health. Robustness checks such as placebo tests and parallel trend tests are conducted. Using PSM-DID estimation, the average treatment effect of in-situ urbanization on health is obtained, and the mechanisms through which these effects occur are explored. This study contributes to empirical research on individual health. Furthermore, by analyzing individual health from the perspective of in-situ urbanization, this study excludes health migration effects and the “salmon bias,” thus obtaining a purer estimate of the health effects of urbanization and effectively addressing longstanding challenges in related research fields.

## 2 Literature review

In-situ urbanization, irrespective of its magnitude, inherently precipitates alterations in the material environment. Consequently, a comprehensive investigation into the health impacts of urbanization necessitates a holistic consideration of changes in living facilities, public services, and the broader transformation of the living environment from a traditional rural setting to an urban landscape. This study, therefore, adopts a broad analytical scope, encompassing all facets of material environmental change associated with in-situ urbanization, as central to understanding its effects on health.

The urban living environment, a direct consequence of urbanization processes, serves as a critical determinant of health outcomes (18). (1) This influence extends beyond the mere physical dimensions of geographic location and material surroundings, encompassing significant socio-economic dimensions as well (11). (2) In developing countries, the impact of the urban living environment on health is particularly pronounced, owing to the substantial disparities between rural and urban areas in terms of infrastructure, health protection, healthcare accessibility, income levels, and social security provisions. The literature identifies three principal theoretical frameworks elucidating the mechanisms through which urbanization affects health:

### 2.1 Social-ecological theory

The social-ecological theory asserts that environmental determinants are pivotal in shaping individual health outcomes. These determinants encompass both the physical and social environments. The physical environment pertains to natural and built environments, while the social environment encompasses socio-economic status, social networks, social capital, and cultural norms (19). Modifications in the physical environment can exert a direct influence on individual health, with variations in infrastructure and ecological conditions potentially leading to disparate health outcomes. Similarly, the social environment prevalent in rural and urban settings can differentially impact health. Urban residents, for instance, may exhibit a lower propensity to assist strangers and possess less extensive familial ties within their social networks compared to their rural counterparts, potentially contributing to variations in interpersonal dynamics and psychological well-being (20). Culture, acting as a fundamental shaper of individual worldviews and health-related behaviors (21), plays a crucial role in influencing both mental and physical health (22). Rural areas, typically characterized by the preservation of more traditional cultural practices than urban areas, exhibit distinct health behaviors compared to urban populations (23).

To further enrich this section, it’s crucial to integrate recent advancements in social-ecological theory. For instance, studies have increasingly emphasized the role of environmental justice in shaping health disparities within urban settings. This perspective highlights how marginalized communities often bear a disproportionate burden of environmental hazards, leading to adverse health outcomes.

Additionally, emerging research has explored the concept of “urbanicity” as a more nuanced measure of urban exposure than simple urban–rural dichotomies. This approach recognizes the heterogeneity within urban areas and examines how different dimensions of urban living (e.g., population density, access to amenities, green space) affect health.

Furthermore, recent work has integrated systems thinking into the social-ecological framework, emphasizing the interconnectedness of various factors influencing health in urban environments. This perspective highlights the importance of considering feedback loops and complex interactions between social, economic, and environmental systems when addressing urban health challenges.

### 2.2 Healthy migrant theory

The healthy migrant theory underscores the health selection effect inherent in migration processes. This perspective posits that individuals who actively engage in migration typically exhibit superior health outcomes compared to non-migrants residing in the destination city (24). The rationale behind this theory is that only individuals possessing robust health are capable of enduring the rigors and challenges associated with migration. Furthermore, various constraints and limitations deter individuals with compromised health from undertaking migratory journeys (25). However, the initial health advantage conferred by this selection effect tends to erode over time as migrants encounter heightened psychological stress and health risks within their new environment (26), coupled with socio-economic disadvantages (27, 44, 45). Moreover, immigrants experiencing health deterioration, often exacerbated by their socio-economic vulnerabilities, face intensified survival pressures, frequently prompting them to return to their original place of residence (27). This phenomenon is commonly referred to as the “salmon bias” (24).

### 2.3 Life-course theory

The life-course theory is frequently employed to analyze the health impacts of urbanization, particularly among older adult populations. This framework emphasizes the enduring influence of environmental exposures and conditions experienced during early life on health and well-being in later years (28, 29). The life-course theory encompasses three primary models: the sensitive-period model, the cumulative-risk model, and the social mobility model (30).

The sensitive-period model postulates that environmental exposures experienced during critical developmental stages in early life exert a disproportionately stronger influence on subsequent health outcomes compared to exposures occurring later in life. The cumulative-risk model focuses on the progressive accumulation of early-life exposures, highlighting their compounding impact on health throughout an individual’s lifespan. The social mobility model proposes that individuals who experience socio-economic disadvantage during early life but subsequently achieve upward mobility benefit from a compensatory effect, leading to improved health outcomes. Conversely, individuals experiencing downward mobility face a penalty effect, resulting in diminished health.

In the context of older adult individuals migrating from rural to urban areas and undergoing urbanization, the life-course theory predicts potential health improvements due to the compensatory effect associated with upward social mobility and improved living conditions.

The relationship between urbanization and health outcomes has been a subject of ongoing scholarly debate. International research has documented associations between urbanization and a spectrum of health risks, including compromised childhood development, increased susceptibility to infectious diseases, elevated mortality rates, heightened prevalence of chronic diseases, and adverse impacts on mental health (31). Concurrently, factors intrinsically linked to urbanization, such as poverty, overcrowding, and environmental degradation, have been shown to exert detrimental effects on health (32). Conversely, urbanization also presents several potential benefits for health. Enhanced employment opportunities, increased income levels, and improved access to educational and healthcare resources can contribute to the amelioration of health outcomes (33).

In China, researchers have conducted extensive empirical investigations into the health effects of urbanization, employing both macro and micro-level analytical approaches. (1) At the macro level, studies utilizing provincial-level panel data have provided insights into the aggregate impacts of urbanization. (2) For instance, Cheng et al. (47), utilizing panel data from 31 Chinese provinces spanning the years 2005 to 2011, demonstrated that increases in urbanization rates were significantly associated with improvements in life expectancy and reductions in infant mortality. Similarly, Jiang et al. (34), analyzing panel data from 30 provinces between 2007 and 2019, identified a non-linear threshold effect between urbanization rates and mortality. (3) Their findings suggested that while urbanization initially contributes to health improvements, this effect diminishes as per capita GDP surpasses a certain threshold.

Recognizing the individual-centric nature of health, a substantial body of research has adopted a micro-level perspective to examine the impact of urbanization on health outcomes. Yan et al. (35), utilizing data from the 2016 and 2018 China Labor Dynamics Survey, revealed that changes in household registration status were associated with improvements in the body mass index (BMI) of migrant workers and a reduction in the probability of being underweight. Furthermore, they observed a decrease in depressive tendencies among migrant workers, indicating positive effects on mental health. Chen et al. (3), drawing on data from the 2014 China Labor Dynamics Survey, identified a non-linear, “U-shaped” relationship between urbanization and economic development and self-reported health. This finding diverges from some studies that reported a linear association (3), highlighting the complexity and potential non-linearity of the relationship between urbanization and individual health.

While a significant portion of research highlights the positive impacts of urbanization on health, it’s important to acknowledge that findings are not uniformly positive. Van et al. (36), utilizing panel data from the China Health and Nutrition Survey (CHNS), constructed an urbanization index based on comprehensive community characteristics and employed a difference-in-differences estimation. Their analysis revealed that urbanization was associated with an increased likelihood of individuals reporting poor health outcomes. Similarly, Wu et al. (37), in their investigation of the interplay between income, healthcare access, and environmental pollution, concluded that the negative health consequences of urbanization, such as environmental degradation, often outweighed the positive effects, including income growth and nutritional improvements. This underscores the nuanced and potentially detrimental aspects of urbanization on individual health.

Beyond the aforementioned micro-level databases, such as the China Labor Dynamics Survey (CLDS), other valuable datasets have been instrumental in facilitating micro-level health studies. Notably, the China Health and Retirement Longitudinal Study (CHARLS) and the China Family Panel Studies (CFPS) have emerged as significant resources for researchers investigating the intricate relationships between socio-economic factors, including urbanization, and individual health outcomes.

Despite extensive research, a definitive consensus on the health effects of urbanization remains elusive. Furthermore, a significant portion of micro-level studies relies on cross-sectional data, limiting the ability to conduct robust longitudinal comparisons. In terms of urbanization modalities, scholarly attention has predominantly focused on traditional migration-based urbanization, while in-situ urbanization has received comparatively less scrutiny.

This paper seeks to address these gaps and contribute to the existing literature by pursuing the following objectives: First, it aims to specifically examine the health impacts of in-situ urbanization, a relatively under-researched area. Second, it employs longitudinal analysis using panel data to investigate the dynamic effects of in-situ urbanization on health. Utilizing data from the China Family Panel Studies (CFPS) for the years 2010, 2012, and 2014, the study employs difference-in-differences (DID) methodologies to compare the health status of individuals before and after in-situ urbanization. Propensity score matching (PSM) is used to ensure the robustness of the findings. Third, the paper aims to elucidate the mechanisms and consequences through which in-situ urbanization influences health outcomes, providing empirical evidence of the positive significance of in-situ urbanization and offering targeted policy recommendations for enhancing public health through urbanization strategies.

## 3 Empirical analysis

### 3.1 Data and variables

#### 3.1.1 Data sources

This study employs data extracted from the China Family Panel Studies (CFPS), a longitudinal survey administered by the Institute of Social Science Survey (ISSS) at Peking University. The CFPS serves as a widely utilized data resource for micro-level empirical research within the domain of health economics. The analytical sample is restricted to individuals aged 16 years and above, utilizing data from the 2010, 2012, and 2014 waves of the CFPS. Notably, the 2012 wave is characterized by the presence of missing information at the community level. Conversely, the 2014 wave incorporates critical survey questions pertaining to the “village-to-residence” (village-to-township) transition within specific village contexts, thereby furnishing essential data for the present investigation into in-situ urbanization. To isolate the health effects of in-situ urbanization on rural residents residing in predominantly rural environments, the study focuses on adult samples drawn from villages located at a substantial distance from central urban areas. The distance between each village and the nearest county town is operationalized as a proxy variable for this spatial dimension.

It is important to note a limitation concerning the 2012 wave of the CFPS data. Specifically, the 2012 wave has limitations in terms of the availability of community-level data. While individual and household-level data remain available, certain community-level variables are not consistently present. This variation in data availability across waves necessitates careful consideration in the empirical analysis.

In this study, our analysis primarily relies on the changes in individual health and household characteristics between 2010 and 2014. While the absence of complete community-level data in 2012 poses a challenge, the difference-in-differences (DID) methodology employed is designed to leverage the longitudinal nature of the data, focusing on changes over time rather than relying heavily on cross-sectional community-level comparisons. Furthermore, we incorporate robustness checks to ensure that our findings are not unduly influenced by the limitations of the 2012 data.

#### 3.1.2 Variables explanation

##### 3.1.2.1 Outcome

The present study investigates the impact of in-situ urbanization on individual health outcomes from a micro-level perspective. Consequently, the primary outcome variable is defined as the individual’s health status. The China Family Panel Studies (CFPS) survey provides a comprehensive set of health-related questions, encompassing both subjective and objective indicators. Subjective health measures include self-reported health assessments, such as “How do you assess your overall health?” and “How do you assess your health compared to others of your age?” Responses to these questions are recorded on a five-point Likert scale. Objective health indicators within the CFPS include anthropometric measures (individual height and weight), reports of recent physical discomfort, hospital visitation records, and chronic disease status.

For the selection of the core outcome variable, this study prioritizes objective health indicators. This decision is predicated on the rationale that in-situ urbanization primarily impacts the material environment, with potentially less direct influence on individuals’ social adaptation. Therefore, objective indicators are deemed more appropriate for capturing the immediate and tangible effects of environmental changes on individual health. Furthermore, given that in-situ urbanization is operationalized through the village-to-residence transition, it is plausible that individuals experience rapid improvements in health due to enhanced living conditions and infrastructure within a few years following the transition.

Accordingly, the core outcome variable selected for this study is “Have you experienced any physical discomfort in the past two weeks?” This variable is binary, with a value of 1 assigned if the respondent reports experiencing physical discomfort within the specified timeframe, and a value of 0 assigned otherwise.

##### 3.1.2.2 Core treatment variable

The core treatment variable in this study is in-situ urbanization. The CFPS village questionnaire includes questions pertaining to the village-to-residence transition, specifically “Has your village implemented the village-to-residence transition?” and “When did the village-to-residence transition occur in your village?” This study utilizes the village-to-residence transition as a proxy measure for in-situ urbanization. To establish a clear temporal distinction between pre- and post-treatment periods, samples from villages that implemented the transition prior to 2011 are excluded. The analytical sample is thus restricted to rural areas where the transition either had not been implemented or was implemented within the period spanning 2011 to 2013. This selection criterion ensures that the treatment group comprises villages undergoing in-situ urbanization during the defined timeframe, while the control group consists of villages that did not experience the transition within this period.

##### 3.1.2.3 Control variables

To ensure a robust assessment of the impact of in-situ urbanization on individual health, and to mitigate potential biases arising from confounding factors, this study incorporates a set of control variables. These variables, encompassing both personal and household characteristics, are selected based on a comprehensive review of the existing literature.

Personal characteristics included in the analysis are: the respondent’s gender, age, years of education, marital status, political affiliation (specifically, membership in the Communist Party of China), and religious beliefs (Table 1).

**Table 1:** Variable description.

Variable name	Meaning	Values
Uncomfort	Whether there was physical discomfort in the past two weeks	Yes = 1, No = 0
Urban	Whether the “village-to-residence” transition has been implemented	Yes = 1, No = 0
Gender	Gender	Male = 1, Female = 0
Age	Age	Age of the respondent
Age2	Age squared	Age of the respondent squared
Edu	Years of education	Duration of education (years)
Marital	Marital status	Married, Cohabiting = 1, Other = 0
Political	Political affiliation	Member of CCP, democratic parties, NPC, CPPCC, union, youth league, women’s federation, or business association = 1, otherwise = 0
Religion	Religious belief	Yes = 1, No = 0
Lincome	Household income	Logarithm of household income
Lwage	Wage income	Logarithm of household wage income
Loperate	Operating income	Logarithm of household operating income
Lproperty	Property income	Logarithm of household property income
Ltransfer	Transfer income	Logarithm of household transfer income
Else	Other income	Logarithm of household other income
Family size	Household size	Number of household members
Toilet	Toilet type	Flush toilet = 1, otherwise = 0
Height	Height	Height of the respondent (cm)
Weight	Weight	Weight of the respondent (kg)
Region	Region	Eastern region = 1, Central/Western region = 0

Household characteristics included in the analysis are: household income and the number of family members.

Preliminary descriptive statistics reveal notable differences between the treatment and control groups. Specifically, the treatment group, representing individuals residing in villages that underwent the village-to-residence transition (in-situ urbanization) between 2011 and 2013, exhibits a lower mean incidence of reported physical discomfort in the preceding two weeks compared to the control group. Conversely, the treatment group demonstrates a higher mean household income. These initial findings provide a valuable reference point for the subsequent empirical analysis (Table 2).

**Table 2:** Descriptive statistics of variables.

Variable	Treatment group			Control group		
	Sample size	Mean	Variance	Sample size	Mean	Variance
Uncomfort	1,486	0.302	0.459	41,562	0.312	0.463
Age	1,486	46.43	16.10	41,562	46.99	15.90
Age2	1,486	2,415	1,512	41,562	2,461	1,518
Gender	1,486	0.480	0.500	41,562	0.490	0.500
Edu	1,486	5.850	4.540	41,562	5.680	4.420
Marital	1,486	0.820	0.390	41,562	0.830	0.370
Politic	1,486	0.130	0.340	41,562	0.130	0.340
Religion	1,486	0.0100	0.0900	41,562	0.0100	0.100
Family size	1,486	4.220	1.610	41,562	4.550	1.950
Lincome	1,486	9.910	1.260	41,562	9.870	1.270
Lwage	1,486	7.310	4.510	41,562	6.990	4.630
Loperate	1,486	5.210	4.190	41,562	6.220	4.010
Lproperty	1,486	1.670	3.270	41,562	0.740	2.270
Ltransfer	1,486	3.680	3.470	41,562	4.130	3.570
Lelse	1,486	1.860	3.340	41,562	1.710	3.100
Toilet	1,467	0.480	0.500	41,110	0.250	0.430

### 3.2 Model design

The implementation of the in-situ urbanization measure, the “village-to-residence” transition, provides a perfect quasi-natural experiment for the study using the Difference-in-Differences (DID) method. Based on panel data from the CFPS surveys conducted in 2010, 2012, and 2014, the sample is divided into treatment and control groups based on whether the village-to-residence transition was implemented. Individuals from villages that implemented the transition make up the treatment group, assigned a value of 1, while individuals from villages where the transition was not implemented make up the control group, assigned a value of 0. Furthermore, based on the specific time of implementation, a period variable is created: pre-village-to-residence transition (=0) and post-village-to-residence transition (=1). An interaction term $\text{urba}n_{\mathrm{ict}}=\text{trea}t_{\mathrm{ic}}\times \mathrm{pos}t_{\mathrm{ct}}$ between the group variable and the period variable is also included [Note: The timing of the village-to-residence implementation differs by location, so the period variable includes these details].^1^ The coefficient of this interaction term measures the net effect of in-situ urbanization on health.

The empirical analysis uses the following DID model:


(1) $$Y_{\mathrm{ict}}=\beta_{1}+\beta_{2}\text{urba}n_{\mathrm{ict}}+\lambda \prime X_{\mathrm{ict}}+\delta_{i}+\gamma_{t}+\varepsilon_{\mathrm{cit}}$$

Here, $Y_{\mathrm{ict}}$ is the dependent variable, Hrepresenting the health status of an individual in the community in year *t*. This study uses objective health indicators to measure individual health, with the corresponding question in the survey being: “Have you had physical discomfort in the past two weeks?” If the answer is “yes,” it is coded as 1; otherwise, it is coded as 0. Compared to self-reported health, which is a relatively subjective indicator, using physical discomfort in the past two weeks provides a more objective and comprehensive reflection of the respondent’s recent physical health.

$\text{urba}n_{\mathrm{ict}}$ The core treatment variable urban is derived from the question: “What year did your community implement the village-to-residence transition?” The information from this question is used to construct the variable. $X_{\mathrm{ict}}$ represents a series of control variables, which include personal characteristics such as age, age squared, gender, years of education, marital status, political affiliation, and religious beliefs. For marital status, unmarried, divorced, or widowed individuals are coded as 0 (no spouse), while married or cohabiting individuals are coded as 1 (with spouse). For political affiliation, individuals who are members of the Communist Party of China, democratic parties, or other organizations such as the People’s Congress, CPPCC, unions, youth league, women’s federation, or business associations are coded as 1, otherwise 0. Individuals who are part of religious groups are coded as 1 for religion, otherwise 0. Household-level control variables include household income and family size. $\delta_{i}$ and $\gamma_{t}$ represent individual fixed effects and time fixed effects, respectively.

- Age and Age Squared: Age is included as health outcomes often have a non-linear relationship with age. We include age squared to capture this potential curvilinear effect, acknowledging that health may decline more rapidly in older age.
- Gender: Gender is controlled for because there are known biological and social differences between men and women that can affect health outcomes. For instance, hormonal differences and societal roles may influence health risks and behaviors.
- Years of Education: Education level is included because it is strongly associated with health literacy, socioeconomic status, and health-related behaviors, all of which can significantly impact health.
- Marital Status: Marital status is included as it has been shown to influence health outcomes. Married or cohabiting individuals (coded as 1) may experience better health due to increased social support, while unmarried, divorced, or widowed individuals (coded as 0) may face different health challenges.
- Political Affiliation: Political affiliation, specifically membership in the Communist Party of China, democratic parties, or other organizations (coded as 1), is considered as it may reflect access to certain resources, social networks, and opportunities that could indirectly affect health.
- Religious Beliefs: Individuals who are part of religious groups (coded as 1) are included as religious beliefs and practices can influence lifestyle choices, coping mechanisms, and social support, all of which can have implications for health.

The prerequisite for using the DID method is that the treatment group and the control group must satisfy the parallel trends assumption, meaning that in the absence of the village-to-residence transition, the health trends of both groups should not exhibit systematic differences over time. However, given real-world differences in regional, social, and policy contexts, this parallel trends assumption may not always hold. Therefore, this study uses the propensity score matching difference-in-differences method to conduct the corresponding robustness check. The idea behind this method originates from matching estimators. The basic approach is to find an individual j in the control group who has not experienced the village-to-residence transition, ensuring that j is as similar as possible to an individual i in the treatment group in terms of observable variables. When the effect of individual characteristics on the likelihood of experiencing the village-to-residence transition depends entirely on observable control variables, the probability of individuals j and i undergoing the transition is similar, allowing for a valid comparison. Based on this score, this study matches the samples and then re-estimates the difference-in-differences regression using only the matched samples within the common support region.

### 3.3 Basic regression analysis

#### 3.3.1 Basic regression results analysis

Table 3 presents the baseline regression results derived from Equation 1. Column (1) of the table displays the results obtained when controlling solely for age and age squared. Column (2) expands the model by incorporating all individual-level control variables, specifically years of education, gender, marital status, political affiliation, and religious beliefs. Column (3) further extends the model to include both individual and household-level control variables, namely household income and family size, while omitting time fixed effects. Finally, Column (4) presents the full model specification, incorporating all individual and household-level control variables, as well as individual and year fixed effects.

**Table 3:** Basic regression results.

Variable	Uncomfort			
	(1)	(2)	(3)	(4)
*In-situ* urbanization	−0.0674***	−0.0680***	−0.0647***	−0.0670***
	(0.0228)	(0.0227)	(0.0227)	(0.0228)
Age	−0.0098	−0.0075	0.0019	−0.0076
	(0.0108)	(0.0109)	(0.0043)	(0.0109)
Age squared	0.0002***	0.0002***	0.0002***	0.0002***
	(0.0000)	(0.0000)	(0.0000)	(0.0000)
Gender		−0.0046	−0.0046	−0.0047
		(0.0899)	(0.0899)	(0.0899)
Education		−0.0073***	−0.0091***	−0.0073***
		(0.0022)	(0.0021)	(0.0022)
Marital status		−0.0255	−0.0259	−0.0257
		(0.0189)	(0.0190)	(0.0190)
Political affiliation		−0.0313***	−0.0298***	−0.0313***
		(0.0114)	(0.0114)	(0.0114)
Religion		0.0235	0.0220	0.0236
		(0.0290)	(0.0290)	(0.0290)
Total income			−0.0052**	−0.0048*
			(0.0025)	(0.0025)
Family size			0.0015	0.0013
			(0.0028)	(0.0028)
Year fixed effects	Yes	Yes	No	Yes
Individual fixed effects	Yes	Yes	Yes	Yes
Sample size	39,855	39,855	39,855	39,855
*R* ^2^	0.2926	0.2930	0.2926	0.2930

***, **, and * represent significance at the 1, 5, and 10% levels, respectively.

The empirical results demonstrate that in-situ urbanization reduces the probability of individuals reporting physical discomfort, thereby improving their physical health status. Specifically, the estimation presented in Column (1) indicates that individuals subjected to the in-situ urbanization policy experienced a 6.7% reduction in the probability of reporting physical discomfort. Furthermore, even with the progressive inclusion of control variables from Column (2) to Column (4), the coefficient remains statistically significant at the 1% level. Consequently, it can be concluded that in-situ urbanization significantly reduces the probability of individuals reporting physical discomfort.

#### 3.3.2 Parallel trend test

Given that individual health status is a categorical variable rather than a continuous numerical variable, a parallel trend test is conducted to validate the difference-in-differences (DID) assumption. The test is performed as follows: First, the 2014 data is excluded from the analysis, and only data from 2010 and 2012 are retained. Among the observations in 2010 and 2012, individuals residing in villages that implemented in-situ urbanization in 2013 are designated as the treatment group. Conversely, individuals from villages that did not implement in-situ urbanization between 2010 and 2012 are assigned to the control group. This process yields a total of 19,854 observations.

The rationale behind this test is to examine whether the treatment and control groups exhibited similar trends in health status changes prior to the implementation of in-situ urbanization. If the regression coefficients associated with the treatment group are statistically insignificant and close to zero, it suggests that the health status trends of the treatment and control groups were indeed parallel before the implementation of in-situ urbanization.

Table 4 presents the regression results obtained using the sample constructed for the parallel trend test. In this context, the treatment group variable, denoted as, is assigned a value of 1 in 2012 and 0 otherwise. Conversely, the control group variable, is assigned a value of 0 in both 2010 and 2012.

**Table 4:** Parallel trend test.

Variable	Uncomfort			
	(1)	(2)	(3)	(4)
*In-situ* urbanization	0.0278	0.0270	0.0261	0.0261
	(0.0453)	(0.0453)	(0.0454)	(0.0453)
Individual control variables	Yes	Yes	Yes	Yes
Family control variables	No	No	Yes	Yes
Year fixed effects	Yes	Yes	No	Yes
Individual fixed effects	Yes	Yes	Yes	Yes
Sample size	19,854	19,854	19,854	19,854
*R* ^2^	0.2952	0.2958	0.2947	0.2958

***, **, and * represent significance at the 1, 5, and 10% levels, respectively.

Column (1) of Table 4 displays the results obtained when controlling solely for age and age squared. Column (2) expands the model by incorporating all individual-level control variables, including years of education, gender, marital status, political affiliation, and religious beliefs. Column (3) further extends the model to include both individual-level and household-level control variables, specifically household income and family size, while omitting year fixed effects. Finally, Column (4) presents the full model specification, incorporating all individual-level and household-level control variables, as well as individual and year fixed effects.

The results presented in Table 4 demonstrate that across all model specifications, the regression coefficients associated with remain statistically insignificant. This finding indicates that, in the absence of the in-situ urbanization shock, the health trends of the treatment group and the control group do not exhibit statistically significant differences. This result provides strong evidence in support of the parallel trends assumption, validating the applicability of the difference-in-differences (DID) methodology in this study.

#### 3.3.3 Placebo test

To address the potential concern that the observed effects of village-to-township urbanization might be attributable to incidental factors rather than the policy itself, a placebo test is conducted. The placebo test is implemented through the following procedure:

First, a random subset of 701 individuals is selected from the control group sample to serve as a pseudo-treatment group. Within this subset, 384 individuals are randomly assigned a hypothetical exposure to the local urbanization policy in 2012, while the remaining 317 individuals are randomly assigned a hypothetical exposure in 2013. A “pseudo-policy dummy variable” is then constructed to represent these randomly assigned exposure times.

Next, the regression analysis is performed using Equation 1, and the pseudo-regression coefficient of the interaction term, along with its corresponding *p*-value, is recorded. This process is repeated 500 times, generating a distribution of pseudo-regression coefficients under the assumption of random policy exposure. This allows for an assessment of whether the observed treatment effect is likely to have arisen by chance.

**Figure 1:**
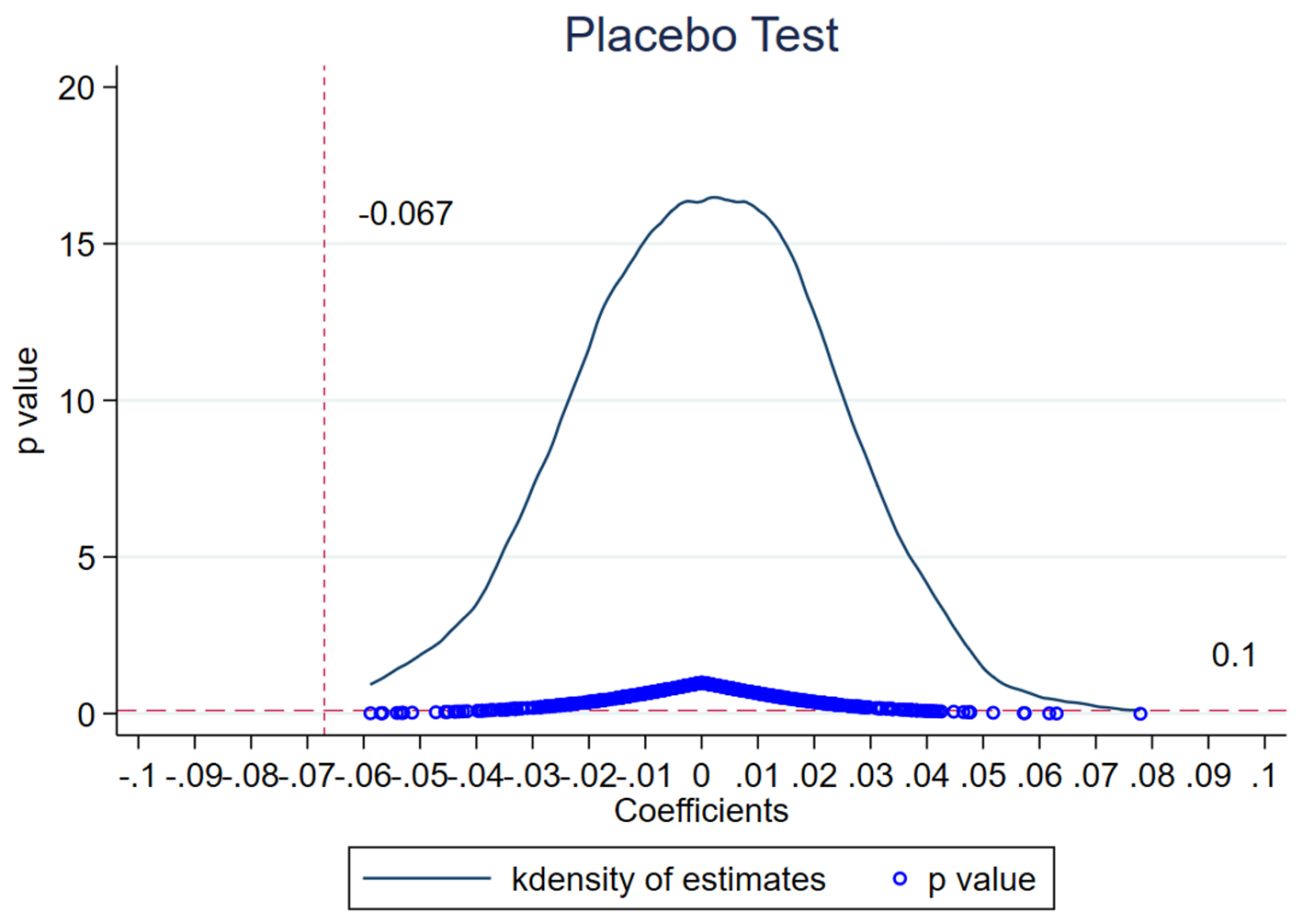
Placebo test.

Figure 1 presents the distribution of the 500 placebo regression coefficients and their corresponding *p*-values generated from the placebo test. As depicted in Figure 1, the 500 placebo regression coefficients are clustered around zero, and all of them are located to the right of −0.067. This implies that all 500 placebo regression coefficients are greater than the coefficient obtained from the baseline regression analysis.

Furthermore, a substantial proportion of the p-values associated with these 500 placebo regression coefficients exceed 0.1, indicating that the majority of the placebo regression coefficients are statistically insignificant.

In summary, the results of the placebo test effectively rule out the possibility that the observed changes in individual health status within the treatment group, as documented in the baseline regression, are attributable to random factors. This provides robust evidence that the observed health improvements are indeed a consequence of the in-situ urbanization policy shock.

### 3.4 Sensitivity analysis

To further strengthen the empirical evidence of this study, the following additional health variables are selected as outcomes: H1, corresponding to the CFPS survey question “How do you assess your overall health?” with responses classified into five categories and assigned values from 1 to 5 in ascending order; H2, corresponding to the question “How does your current health compare to one year ago?” with responses also classified into five categories and assigned values from 1 to 5 in ascending order; three dummy variables, including medical treatment (assigned 1 if the respondent was hospitalized in the past 12 months, otherwise 0), smoking (assigned 1 if the respondent smoked in the past month, otherwise 0), and drinking (assigned 1 if the respondent consumed alcohol more than three times per week in the past month, otherwise 0); and BMI, representing the individual’s body mass index, calculated based on height and weight.^2^

The regression results presented in Table 5 demonstrate that the coefficients associated with the two self-reported health indicators, H1 and H2, as well as BMI, are all positive. Notably, the coefficient for self-reported health H2 achieves statistical significance at the 10% level. These findings suggest that in-situ urbanization contributes to improvements in individual health, which aligns with the conclusions drawn from the baseline model. Furthermore, the results indicate a marginal decrease in unhealthy behaviors, specifically excessive alcohol consumption. While these results do not reach statistical significance, they offer supplementary evidence that is relevant to the study’s overall findings.

**Table 5:** The impact of *in-situ* urbanization on different health indicators.

Variable	H1	H2	Medical visit	Smoking	Drinking	BMI
	(1)	(2)	(4)	(5)	(6)	(7)
*In-situ* urbanization	0.0536	0.0327*	0.0192	0.1742	−0.0187	0.1164
	−0.0518	−0.0176	−0.0158	−0.4115	−0.0144	−0.1103
Control variables	Yes	Yes	Yes	Yes	Yes	Yes
Year fixed effects	Yes	Yes	Yes	Yes	Yes	Yes
Individual fixed effects	Yes	Yes	Yes	Yes	Yes	Yes
Sample size	39,855	39,854	39,851	39,717	39,799	37,297
*R* ^2^	0.5612	0.1362	0.156	0.5829	0.5554	0.715

**, **, and * represent significance at the 1, 5, and 10% levels, respectively.

### 3.5 Robustness check

Building upon the preceding tests, this study conducts additional robustness checks to further validate the findings. As presented in Table 6, Column (1) displays the estimation results obtained after trimming the top and bottom 5% of continuous variables, including age, years of education, and household income, thereby mitigating the influence of extreme values. Column (2) excludes samples that have experienced land requisition, as this process often introduces stress and uncertainty among local residents, potentially affecting their physical and mental well-being. Column (3) removes samples that have undergone demolition, given that demolition typically involves disruptions to living environments and social networks, which can confound the assessment of residents’ health. Column (4) refines the distance threshold from the sample village to the nearest county town, setting it at 20 kilometers, to better control for location-specific factors that may influence health.

**Table 6:** Robustness check.

Variable	Uncomfort			
	(1)	(2)	(3)	(4)
*In-situ* urbanization	0.0669***	0.0746***	0.0697***	0.0738**
	(0.0228)	(0.0238)	(0.0232)	(0.0330)
Control variables	Yes	Yes	Yes	Yes
Year fixed effects	Yes	Yes	Yes	Yes
Individual fixed effects	Yes	Yes	Yes	Yes
Sample size	39,855	35,799	39,220	26,835
*R* ^2^	0.2931	0.2986	0.2953	0.2977

***, **, and * represent significance at the 1, 5, and 10% levels, respectively.

The estimation results presented in Columns (1) through (4) are consistently negative, with magnitudes generally aligned with those observed in the baseline model, and all reach statistical significance at the 1% level. These findings provide strong evidence that the estimation results derived from the baseline model are robust and resilient to various potential confounding factors.

### 3.6 Propensity score matching and difference-in-differences (PSM-DID) estimation

The validity of the difference-in-differences (DID) model relies on the assumption of random assignment. However, in the context of in-situ urbanization, the implementation of this policy at the village level is not random. It is influenced by a range of factors, including geographical location and factor agglomeration, with rural areas proximate to cities or small towns exhibiting a higher propensity for in-situ urbanization. Consequently, an individual’s exposure to the in-situ urbanization policy shock is determined by their residential location, rather than through a process of random assignment.

The application of the DID model to estimate the impact of in-situ urbanization on individual health in the presence of non-random selection is susceptible to selection bias. To mitigate the endogeneity issue arising from this non-random selection, this study employs the propensity score matching difference-in-differences (PSM-DID) method. Specifically, cross-sectional matching using k-nearest neighbor matching is utilized to construct comparable treatment and control groups. Subsequently, a difference-in-differences analysis is performed on these matched groups, effectively addressing the potential bias introduced by non-random selection.

#### 3.6.1 Balance test

Propensity score matching (PSM) relies on the common support assumption, which necessitates the exclusion of samples that do not satisfy this assumption after the matching process. However, excessive exclusion of samples can lead to a substantial reduction in the regression sample size, potentially compromising the accuracy of the final estimates. Therefore, to ascertain the quality of the data utilized in the difference-in-differences (DID) regression following PSM, a balance test is essential prior to conducting the DID analysis.

The balance test aims to verify whether there are statistically significant differences in the means of the covariates between the treatment and control groups after the matching procedure. This test ensures that the PSM effectively creates comparable treatment and control groups, mitigating potential biases that could arise from imbalances in pre-treatment characteristics.

Table 7 presents the distribution of all matching variables utilized in the propensity score matching procedure, specifically post-matching. As demonstrated in Table 7, the standardized biases for all matched variables exhibit a substantial reduction following the matching process, with all biases falling below 10%, a value significantly lower than the pre-matching biases. Furthermore, the t-test results for all variables post-matching are not statistically significant at the 10% level, indicating a failure to reject the null hypothesis of no systematic differences between the treatment and control groups. This analysis confirms the effectiveness of the propensity score matching process and its successful passage of the balance test.

**Table 7:** Balance test of control variables before and after PSM.

Variable	Unmatched (U)	Mean		%Bias	%Reduct |bias|	*t*-test	
	Matched (M)	Treatment group	Control group			*t*	*p* > |*t*|
Age	U	46.184	46.982	−4.9	59.4	−1.18	0.236
	M	46.184	45.86	2.0		0.33	0.741
Age squared	U	2403.2	2460.1	−3.7	50.6	−0.89	0.376
	M	2403.2	2,375	1.8		0.3	0.761
Gender	U	0.4885	0.48935	−0.2	−595.0	−0.04	0.968
	M	0.4885	0.48257	1.2		0.20	0.842
Education	U	5.8956	5.6873	4.6	86.8	1.11	0.267
	M	5.8956	5.9231	−0.6		−0.10	0.919
Marital status	U	0.80708	0.83233	−6.6	52.7	−1.60	0.111
	M	0.80708	0.79513	3.1		−0.50	0.615
Political affiliation	U	0.12212	0.13344	−3.4	−0.9	−0.79	0.432
	M	0.12212	0.13354	−3.4		0.57	0.566
Religion	U	0.01239	0.01073	1.5	46.6	0.38	0.705
	M	0.01239	0.01150	0.8		0.14	0.891
Total income	U	10.052	9.8669	14.5	95.3	3.45	0.001
	M	10.052	10.044	0.7		0.12	0.905
Family size	U	4.292	4.5438	−13.9	93.6	−3.07	0.002
	M	4.292	4.2758	0.9		0.16	0.873

**Figure 2:**
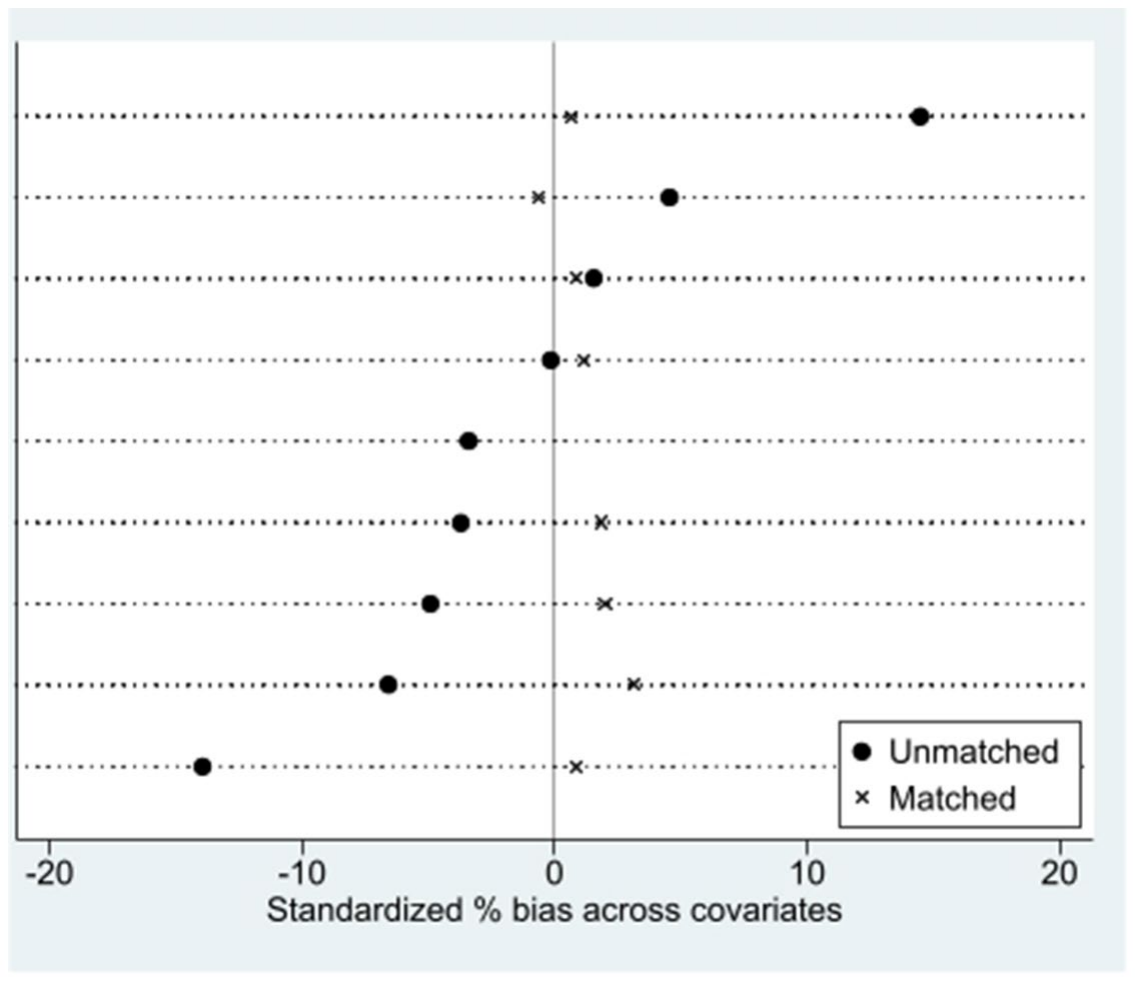
Changes in standardized bias of control variables.

Figure 2 provides a visual representation of the changes in the standardized bias of each matched variable before and after the propensity score matching (PSM) process. The solid black circles in the figure denote the standardized biases of the variables before matching, while the “×” marks represent the standardized biases of the variables after matching. As clearly depicted in Figure 2, the standardized bias for all variables exhibits a reduction following the matching procedure, and all biases are contained within the ±10% range. This visual evidence reinforces the quantitative findings of the balance test, demonstrating the effectiveness of the PSM in creating well-balanced treatment and control groups.

#### 3.6.2 Analysis of PSM-DID estimation results

Following the preceding analysis, the matching outcomes derived from k-nearest neighbor matching successfully passed the balance test, demonstrating the absence of statistically significant differences between the treatment and control groups across all variables. Subsequently, this study employs the matched samples to conduct difference-in-differences (DID) estimation. Table 8 presents a comparative analysis of the DID and PSM-DID estimation results. Specifically, Column (1) displays the DID estimation without control variables, Column (2) presents the DID estimation with all control variables, Column (3) shows the PSM-DID estimation without control variables, and Column (4) provides the PSM-DID estimation with all control variables. The results presented in Table 8 indicate a high degree of consistency between the estimates obtained using both the DID and PSM-DID methods, suggesting the robustness of the DID estimation results. The analysis confirms that in-situ urbanization significantly improves individual health, resulting in an approximate 6.7% reduction in the probability of individuals reporting physical discomfort.

**Table 8:** Comparison of DID and PSM-DID estimation results.

Variable	Uncomfort			
	DID		PSM-DID	
	(1)	(2)	(3)	(4)
*In-situ* urbanization	−0.0687***	−0.0670***	−0.0691***	−0.0671***
	(0.0228)	(0.0228)	(0. 0228)	(0.0228)
Control variables	No	Yes	No	Yes
Year fixed effects	Yes	Yes	Yes	Yes
Individual fixed effects	Yes	Yes	Yes	Yes
Sample size	39,855	39,855	39,310	39,310
*R* ^2^	0.5637	0.2926	0.5642	0.2926

***, **, and * represent significance at the 1, 5, and 10% levels, respectively.

### 3.7 Heterogeneity analysis

The “14th Five-Year Plan for New-Type Urbanization Implementation Plan,” promulgated by the National Development and Reform Commission, explicitly articulates the objective of promoting the equalization of basic public services and achieving a substantial improvement in the quality of urbanization for the rural-to-urban migrant population by 2025. To provide a more comprehensive and nuanced assessment of the impact of in-situ urbanization on health improvements across diverse population segments, this study conducts subgroup analyses based on gender, age, and years of education, employing the following regression model (Equations 2–4).


(2) $$\begin{array}{l} Y_{\mathrm{ict}}=\beta_{1}+\beta_{2}(\text{urba}n_{\mathrm{ict}}\times 1(\text{gender}=1\;\text{or}\;0))\\ +\sum \beta_{i}X_{\mathrm{ict}}+\delta_{i}+\gamma_{t}+\varepsilon_{\mathrm{cit}} \end{array}$$

(3) $$\begin{array}{l} Y_{\mathrm{ict}}=\beta_{1}+\beta_{2}(\text{urba}n_{\mathrm{ict}}\times 1(\mathrm{age}<\text{or}\geq 60))\\ +\sum \beta_{i}X_{\mathrm{ict}}+\delta_{i}+\gamma_{t}+\varepsilon_{\mathrm{cit}} \end{array}$$

(4) $$\begin{array}{l} Y_{\mathrm{ict}}=\beta_{1}+\beta_{2}(\text{urba}n_{\mathrm{ict}}\times 1(\mathrm{edu}<\text{or}\geq 9))\\ +\begin{array}{c} \sum \beta_{i}X_{\mathrm{ict}}+\delta_{i}+\gamma_{t}+\varepsilon_{\mathrm{cit}}\# \end{array} \end{array}$$

Table 9 presents the results of the subgroup analyses. Columns (1) and (2) reveal that the health-enhancing effect of in-situ urbanization is more pronounced among females. This disparity may be attributed to the generally poorer health status of women engaged in agricultural labor compared to men, making them more susceptible to the positive health impacts of material environmental improvements brought about by in-situ urbanization.

**Table 9:** Heterogeneity analysis.

Variable	Uncomfort					
	Gender		Age		Education	
	(1) Male	(2) Female	(3) Over 60	(4) 16 ~ 60 years old	(5) Below high school	(6) Above high school
*In-situ* urbanization	−0.0458	−0.0856***	−0.0495*	−0.1055*	−0.0820***	−0.0174
	(0.0317)	(0.0326)	(0.0253)	(0.0550)	(0.0252)	(0.0584)
Control variables	Yes	Yes	Yes	Yes	Yes	Yes
Year fixed effects	Yes	Yes	Yes	Yes	Yes	Yes
Individual fixed effects	Yes	Yes	Yes	Yes	Yes	Yes
Sample size	19,409	20,400	29,445	8,859	34,716	4,274
*R* ^2^	0.2745	0.2936	0.2914	0.2485	0.2923	0.2752

***, **, and * represent significance at the 1, 5, and 10% levels, respectively.

Columns (3) and (4) demonstrate that in-situ urbanization significantly improves health outcomes for both individuals aged 16–60 and those over 60. However, the health improvement effect is substantially stronger for the 16–60 age group. This difference may stem from the higher likelihood of individuals over 60 exiting the labor force and becoming dependent older adult, thus experiencing less direct income impact from in-situ urbanization.

Columns (5) and (6) indicate that in-situ urbanization has a greater health-enhancing effect on individuals with lower educational attainment. This variation in health improvement effects may be due to differences in occupational transitions. Compared to individuals with a high school education or higher, those with lower education levels were more likely to be engaged in agricultural work. Following the implementation of in-situ urbanization, individuals with lower education levels may experience a more significant shift from agricultural to non-agricultural employment, leading to greater improvements in physical health compared to their higher-educated counterparts who were already more likely to be in non-agricultural sectors.

### 3.8 Intermediate mechanism: the income effect of *in-situ* urbanization

The rapid development of health economics, largely attributable to Grossman’s seminal work on health demand, has spurred extensive theoretical and empirical investigations into the determinants of health. It is widely acknowledged that health is influenced by a complex interplay of factors, including healthcare access, income levels, educational attainment, occupational characteristics, age, gender, marital status, and numerous other variables. While prior research has established the positive impact of in-situ urbanization on individual health, the specific mechanisms through which this effect operates remain unclear.

This study seeks to elucidate the intermediate mechanisms underlying the health-enhancing effects of in-situ urbanization. Following Jiang (34) recommendation, this paper refrains from a step-by-step mediation analysis and instead identifies intermediate variables, grounded in established economic theories, that reflect the potential pathways through which in-situ urbanization influences health outcomes. Subsequently, the baseline model is employed to establish the causal relationship between in-situ urbanization and these intermediate variables.

In health economics, “health” exhibits characteristics of both an investment and a consumption good, rendering the relationship between income and health particularly complex. From a theoretical standpoint, income serves as a pivotal independent variable within Grossman’s health demand function. Higher income levels typically correlate with improved living conditions, dietary habits, access to healthcare services, and other health-enhancing factors.

Empirical research has consistently demonstrated a causal link between income and health, spanning macro-level analyses (38) and micro-level investigations (39), as well as studies focusing on absolute (40) and relative (41) income effects. In high-income countries, the impact of income on health is generally positive, albeit its magnitude may vary depending on contextual factors. In low- and middle-income countries, the relationship between income and health is especially pronounced. As income increases in these countries, it facilitates greater consumption of survival-related goods and services, leading to reductions in infant mortality and yielding substantial health benefits (16). Consequently, this paper prioritizes the examination of the impact of in-situ urbanization on income as a potential mechanism through which health improvements are realized.

To empirically investigate the impact of in-situ urbanization on income, this study estimates both total household income and its structural components, including wage income, transfer income, business income, property income, and other income, using the baseline model. To enhance the reliability and comparability of the results, the income data for 2012 and 2014 are adjusted to 2010 constant terms. This adjustment ensures that the income data reflects real changes over time, rather than nominal fluctuations due to inflation. The following model is estimated (Equation 5):


(5) $$W_{\mathrm{ict}}=\beta_{1}+\beta_{2}\text{urba}n_{\mathrm{ict}}+\sum \beta_{i}X_{\mathrm{ict}}+\delta_{i}+\gamma_{t}+\varepsilon_{\mathrm{cit}}$$

Where $W_{\mathrm{ict}}$ represents income variables, including total household income, wage income, transfer income, property income, operating income, and other income (all in logarithmic form). The estimation results are shown in Table 10. Column (1) indicates that, overall, in-situ urbanization increases household income by 19.15%. Columns (2) to (6) show that in-situ urbanization significantly increases business and property income. On the one hand, the implementation of village-to-township urbanization may activate idle rural land, which could increase cultivated land and, in turn, agricultural production income. On the other hand, due to the potential advantages in location-based factor agglomeration, income from individual businesses may also increase after in-situ urbanization. Furthermore, the implementation of village-to-township urbanization and the potential changes in land use rights may lead to more population and factor agglomeration, which will further increase household property income.

**Table 10:** The income effect of *in-situ* urbanization.

Variable	Lincome	Lwage	Ltransfer	Loperate	Lproperty	Lelse
	(1)	(2)	(3)	(4)	(5)	(6)
*In-situ* urbanization	0.1915***	0.2131	−0.3416**	0.6553***	0.8586***	−0.2284
	(0.0574)	(0.1796)	(0.1517)	(0.1685)	(0.1341)	(0.1572)
Control variables	Yes	Yes	Yes	Yes	Yes	Yes
Year fixed effects	Yes	Yes	Yes	Yes	Yes	Yes
Individual fixed effects	Yes	Yes	Yes	Yes	Yes	Yes
Sample size	39,855	39,855	39,855	39,855	39,855	39,855
*R* ^2^	0.3927	0.3844	0.5176	0.4431	0.3822	0.1458

***, **, and * represent significance at the 1, 5, and 10% levels, respectively.

The results presented in Column (3) indicate that in-situ urbanization leads to a statistically significant reduction in household transfer income. This reduction in transfer income, which primarily comprises government subsidies, may be attributed to the improved economic conditions of households following in-situ urbanization. As households experience increased income and economic stability, their reliance on government subsidies may decrease.

Furthermore, the analysis reveals that in-situ urbanization has a relatively limited impact on wage income and other income sources. This suggests that while in-situ urbanization influences the structure of household income, particularly by reducing reliance on transfer payments, its effects on wage income and other income streams are less pronounced.

A defining characteristic of urbanization is the shift from agricultural to non-agricultural employment. (1) This transition was prominent during the initial phases of in-situ urbanization in the Jiangsu and Zhejiang regions (49), marked by a substantial increase in wage income. Notably, over 70% of the communities undergoing in-situ urbanization are situated in the less developed central and western regions of China. In contrast, in the economically advanced eastern region, in-situ urbanization is often accompanied by the proliferation of industrial enterprises and accelerated economic development at the county level (49). Consequently, farmers in these areas experience greater ease in transitioning to non-agricultural occupations, resulting in a significant augmentation of wage income as a proportion of their total income.

Conversely, in the central and western regions of China, the transition to non-agricultural employment is constrained by limitations in geographical location, industrial development, and county-level economic advancement, resulting in a scarcity of non-agricultural job opportunities. In these regions, in-situ urbanization is frequently driven by top-down government initiatives, and the division of labor among workers is less specialized (51). Consequently, households in these areas do not experience a substantial increase in wage income following in-situ urbanization. This regional disparity highlights the importance of considering local economic contexts when assessing the impact of in-situ urbanization on employment and income.

## 4 Conclusion and policy recommendations

### 4.1 Research conclusions

Using longitudinal data from the 2010, 2012, and 2014 China Family Panel Studies (CFPS) tracking surveys, this paper employs the difference-in-differences (DID) model to examine the health effects of in-situ urbanization, leading to the following conclusions:

Significant Health Improvement: In-situ urbanization significantly enhances individual health. Individuals exposed to in-situ urbanization experience a 6.7% reduction in the probability of reporting physical discomfort within the past two weeks, a result statistically significant at the 1% level. This finding is robust, as evidenced by the successful passage of parallel trend and placebo tests, as well as robustness checks involving the exclusion of samples affected by land requisition or demolition, the treatment of outliers in continuous variables, and modifications to the sample’s distance to the county. Furthermore, the robustness of the results is confirmed through the application of the propensity score matching difference-in-differences (PSM-DID) method.

Heterogeneous Health Effects: The health effects of in-situ urbanization exhibit heterogeneity across different demographic groups. Specifically, females experience a greater health improvement effect compared to males. Individuals aged 60 and below demonstrate significant health improvements, while the effect is not significant for those over 60. Moreover, individuals with lower educational attainment (below high school) experience stronger health improvement effects.

Income as a Mechanism: In-situ urbanization improves individual health by augmenting household income. Overall, in-situ urbanization leads to a 19.15% increase in household income, a finding statistically significant at the 1% level. Structurally, business and property income increase following in-situ urbanization, while transfer income decreases. Consequently, the increase in business and property income constitutes a crucial intermediate mechanism through which in-situ urbanization enhances health outcomes.

While this study provides valuable insights into the health effects of in-situ urbanization in China, future research could explore the long-term health impacts of this process. Longitudinal studies that track individuals over an extended period would provide a more comprehensive understanding of the relationship between in-situ urbanization and health outcomes.

This study focused on the average effects of in-situ urbanization. Future research could investigate the heterogeneous effects of in-situ urbanization on specific subgroups of the population, such as the older adult or individuals with chronic diseases. This would help to identify vulnerable populations and inform targeted interventions.

Our analysis relied on CFPS data. Future studies could utilize other data sources, such as remote sensing data or local health records, to validate our findings and provide a more comprehensive assessment of the health impacts of in-situ urbanization.

This research examined the impact of in-situ urbanization on physical health. Future studies could also explore the effects of in-situ urbanization on mental health and social well-being.

Given the regional variations in in-situ urbanization, future research could conduct comparative studies across different regions in China to examine how local contexts influence the health effects of urbanization.

By adding a section on future research directions, you can demonstrate that your study contributes to an ongoing conversation and opens up new avenues for inquiry.

### 4.2 Policy recommendations

#### 4.2.1 Scientifically choose urbanization paths in the new development stage

In-situ urbanization and migration-based urbanization, as distinct approaches to urbanization, do not possess an inherent absolute advantage over one another. Rather, their respective roles are contingent upon the specific stage of urbanization development. During the nascent stages of urbanization, rapid shifts in industrial structure and the proliferation of industrial and commercial enterprises generate a substantial and immediate demand for labor. This demand cannot be adequately addressed solely through in-situ urbanization. Concurrently, the higher wages offered by the industrial sector, relative to the agricultural sector, incentivize rural laborers to migrate to central cities or even distant developed urban centers, thereby facilitating migration-based urbanization and driving a rapid increase in urbanization rates. This pattern represents the successful urbanization trajectory observed in China over the past 40 years, as well as in Western developed countries since the Industrial Revolution.

Currently, China’s urbanization rate has reached nearly 64%, signifying its transition into an advanced stage of urbanization. (1) While further urbanization is possible, replicating the high-speed growth model of the past is challenging. Simultaneously, China’s economy has entered a new phase, prioritizing high-quality growth. (2) The industrial structure in the eastern developed regions, which historically absorbed surplus rural labor and facilitated migration-based urbanization, is undergoing continuous optimization. Sectors such as education, finance, and technology are now the primary drivers of economic growth, while low-end manufacturing industries, which previously absorbed large numbers of rural laborers, are gradually being phased out. Some of these industries have relocated to the central and western regions, while a substantial portion has shifted to Southeast Asia and India, where labor costs are even lower. This indicates that the economic conditions that previously supported migration-based urbanization are no longer as favorable. Consequently, in-situ urbanization should be positioned as the primary strategy for advancing urbanization in China moving.

#### 4.2.2 Inject new economic vitality through *in-situ* urbanization

Traditional migration-based urbanization has not only facilitated the rapid transition of a significant labor force from agricultural to non-agricultural employment but has also fueled continuous urban development. From the “4 trillion stimulus plan” implemented in 2008 to the “Three Reductions and One Supplement” policy introduced in 2015, infrastructure investment, including urban construction, has long served as a crucial engine for economic growth. However, with the saturation of traditional infrastructure projects, special bonds, which primarily finance investment initiatives, are facing a scarcity of viable projects, thereby exacerbating the issue of insufficient effective investment demand.

Indeed, small towns serve as the cornerstone of in-situ urbanization, presenting substantial investment opportunities. China’s existing network of over 20,000 townships, with an average population of 9,000, possesses significant expansion potential (43). If each township were to accommodate an average population of 30,000, it would create an urbanization capacity for approximately 420 million people. This transformation translates to an estimated annual direct investment demand of around 2 trillion yuan (42), generating substantial economic benefits. This highlights the vital role of small towns in driving economic growth and urbanization in China.

#### 4.2.3 Human-centered urbanization

In the continued pursuit of urbanization, a human-centered approach is paramount. While traditional migration-based urbanization offers increased employment and income opportunities, it also poses significant health risks due to urban challenges such as traffic congestion, air pollution, and overcrowding. In contrast, in-situ urbanization has the potential to create more livable environments, reduce the overall cost of urbanization, and foster healthier urban settings that are more conducive to individual well-being.

#### 4.2.4 Focus on vulnerable groups in the urbanization process

While urbanization offers numerous benefits, its impacts are not uniformly distributed across all population groups. This study’s findings reveal that the health improvement effects of in-situ urbanization are limited for older adult individuals over 60 years of age. Therefore, in the pursuit of in-situ urbanization, it is imperative to prioritize the needs of vulnerable groups. This can be achieved by ensuring equitable access to public services, strengthening the social security system, and enhancing the protection of these groups’ health. This targeted approach will contribute to a more inclusive and equitable urbanization process.

#### 4.2.5 Support resident income growth through industry

A fundamental aspect of urbanization is the shift from agricultural to non-agricultural employment, which can significantly boost residents’ income and provide a solid foundation for improved health outcomes. However, this study reveals that in-situ urbanization in the central and western regions, often driven by top-down government initiatives, has limited impact on increasing wage income. Therefore, towns undergoing in-situ urbanization should prioritize the development of regional industries. This can be achieved by leveraging local advantages and unique resources to promote advanced manufacturing, transportation hubs, commercial trade, and cultural tourism, thereby creating more non-agricultural job opportunities and supporting resident income growth.

## Data Availability

Publicly available datasets were analyzed in this study. This data can be found at: CFPS, www.isss.pku.edu.cn/cfps.

## Footnotes

^1^ http://www.gd.gov.cn/zwgk/gongbao/1985/7/content/post_3354518.html

^1^Since the timing of the village-to-residence transition varies across different locations, the subscript c is included in the period variable.

^2^BMI is calculated as weight (kg) divided by the square of height (m).
